# Comparison of Two Types of Preparation for Laminate Veneer with Three Types of All-Ceramic Materials

**DOI:** 10.1055/s-0042-1743143

**Published:** 2022-07-12

**Authors:** Salah A. Yousief, Rami M. Galal, Hassan Mehsen Ahmad Alsharief, Thamer Thyeab R. Alharbi, Khaled Aqeel A. Alzhrani, Husam Talat A. Migaishet, Adil Ahmed A. Alshehri, Ayoub Ismail A. Nouruldeen, Ghaida Ali S. Sait, Yousef Jayar, Reham Alharthi, Sulafah Abdullah Alotaibi

**Affiliations:** 1 Department of Restorative and Prosthetic Dental Sciences, College of Dentistry, Dar Al Uloom University, Riyadh, Saudi Arabia; 2 Crown and Bridge Department, Faculty of Oral and Dental Medicine, Al Azhar University, Assuit Branch, Egypt; 3 Fixed and Removable Prosthodontics Department, National Research Centre, Giza, Egypt; 4 Fixed Prosthodontics Department, AlNahda University, Egypt; 5 Private Practice, Egypt

**Keywords:** Lava Ultimate, IPS e-max, Celtra

## Abstract

**Objective** The objective of this study was to compare types of veneer preparations and their combination with three materials.

**Materials and Methods** Two finite element models were specially prepared used representing window and wrap around preparation for veneers. The “central incisor” tooth geometry was acquired using a laser scanner, and then its surface was adjusted to form a solid model prior to the removal of each preparation separately. Three materials (Lava Ultimate, IPS e-max, and Celtra) were tested in combination with the preparation type. Bone geometry was simplified as two coaxial cylinders in all models. Each model was subjected to two loading conditions of occlusion (edge-to-edge bite and normal bite).

**Statistical Analysis and Results** It was observed that cortical, cancellous bone, and periodontal ligament are insensitive to preparation or materials. Their stresses and deformation were within physiological limits. Significant changes appeared on the central incisor tooth structure, cement layer, and veneer layer stresses and deformations under loading cases.

**Conclusions** Edge-to-edge bite stresses are severe with window-type preparation, and normal bite did not show any critical values on tooth structure, cement layer, or veneer layer. Veneer layer finish line and its contact with the cement layer and tooth structure play a role in the loading transfer mechanism. Preparation type alters the values of stresses on tooth structure, cement, and veneer layers. With window preparation, extreme stresses appear at finish line, while stresses appear under the loading site with wrap around preparation. Veneer and cement layers withstand the load energy with wrap around preparation and reduce tooth structure stresses. Thus, the lifetime of veneer and cement layers might be longer with window preparation.

## Introduction

The ceramic veneers are requested by many patients as they are used for esthetic purposes with minimal tooth preparation that could be only in enamel, slight preparation is done to give thickness to the veneer to assure its strength and color. 1 The ceramic veneers have advantages over direct veneers of much less possibility of discoloration or development of recurrent caries under them. 2 Indirect veneers have limitations and concerns because of the material used in fabrication and the bonding system. In addition, they are affected by parafunctional habits such as bruxism. The main failures of these types of restorations include fractures and dislodgement. The mechanical states and behavior of fixed prostheses can be assessed using finite element analysis. 3 Three common types for laminate veneer preparations are as follows: (1) window or contact lens, (2) classic or conventional, and (3) wrap around or 3/4th type. In the window type, there is no preparation of the incisal edge, it is indicated when teeth have enough length. The wrap around is indicated mainly when there is a need to modify the incisal length or translucency. 4 Some studies investigated the stress distribution of veneers with different designs; finite element analysis showed that joint or classic preparation tolerates stress better, but overlap wrap around distributes stresses more uniformly. The window type causes stress concentration at the incisal area. 5 In meta-analysis of these studies, it was suggested that the overlap type is more subjected to fracture than the window type. 6 Few studies investigated the effect of incisal overlapping on prognosis. Survival rates between covering the incisal edge or not showed no difference in a 2.5 years study. 7 Resin matrix ceramics are a group of prosthetic materials combining the properties of polymers having a modulus of elasticity of dentin but reinforced with ceramics. 8 One type of these materials is the resin nanoceramic containing zirconia nanoparticles, with zirconia silica nanoclusters linked with high-cured resin matrix (bisphenol A glycol dimethacrylate, urethane dimethacrylate, ethoxylated bisphenol A glycol dimethacrylate, and triethylene glycol dimethacrylate). 9 Resin nanoceramic has 12 GPa dentin-like modulus of elasticity and high flexural strength (approximately 150 Mpa) and fracture toughness (approximately 1.2 Mpa m ^1/2^ ). 10 11 12 The use of different materials (resin nanoceramic “Lava Ultimate,” lithium disilicate glass ceramic “IPS e-max” and zirconia reinforced Lithium silicate “Celtra Duo”) is investigated in this study under two loading conditions using finite element analysis. The null hypothesis is that the type of preparation which has an effect on the stresses affecting the veneer and underlying tooth. It is a new study idea to evaluate the effects of different preparation types with three different ceramic materials.

## Materials and Methods

**Fig. 1:**
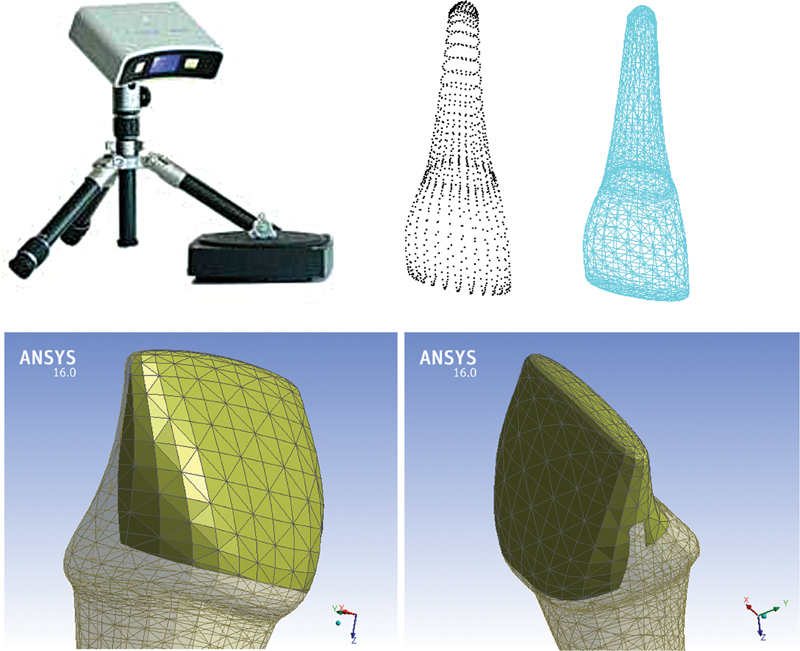
Laser scanner and scanned tooth as cloud of points and after creating its surface.

Two three-dimensional (3D) models for central incisor were prepared for this study. Tooth “central incisor” geometry was acquired using a laser scanner (Geomagic Capture, 3D Systems, Cary, NC, United States) of a sample plastic tooth. The scanner produced data file containing a cloud of point coordinates, as presented in Fig. 1 . An intermediate software was required (Rhino 3.0, McNeel Inc., Seattle, WA, United States) to trim a newly created surface by the acquired points. Then, the solid (closed) tooth geometry was exported to the finite element program in the STEP file format.

As presented in Fig. 1 , the two types of preparations (window and wrap around) were removed using Boolean operations. The geometric configurations of the laminate veneer preparation designs and their dimensions simulating a clinical preparation protocol were introduced into the ANSYS software program (0.5 mm buccal and proximal reduction plus 0.1 mm for cement layer, cervical margin placed 1.0 mm away from the cemento-enamel junction, and 0.5 mm chamfer made for all finish lines).

Bone geometry was simplified and simulated as two coaxial cylinders, with the inner one representing the spongy bone with 14 mm diameter and 22 mm height, filling the internal cylindrical space of the other cylinder (shell of 1 mm thickness) that represented cortical bone (outer diameter of 16 mm and its height of 24 mm). A set of Boolean operations was used to create a root cavity in bone and create a periodontal ligament (PDL) layer.

Three restoration materials were tested in this study: Lava Ultimate, IPS e-max, and Celtra. All materials used in this study were assumed isotropic, homogeneous, and linearly elastic (their properties are listed in Table 1 ). Each of the model components (bone, tooth structure, etc.) was assigned to material properties on the finite element package.

**Table 1:** Material properties used in analysis

	Young's modulus [MPa]	Poisson's ratio
Cortical bone	18,800	0.30
Cancellous bone	10,700	0.30 14
Periodontal ligament	69	0.45 15
Enamel	84,100	0.33 14
Dentin	14,700	0.31 14
Pulp	2	0.45 13
Resin cement	6,000	0.30 1
Lava Ultimate	12,770	0.47
IPS e-max	95,000	0.22 3
Celtra	107,000	0.22

The final models were meshed by brick element that has three degrees of freedom as translations in the global directions on the finite element package ANSYS version 16 (ANSYS Inc., Canonsburg, PA, United States). Adequate mesh density was selected to ensure results' accuracy for the discrete model. Mesh density is another relevant parameter. As the geometries are complex, increasing the mesh density improves the results' accuracy for the discrete model. Another effect of increasing the number of elements is the reduction in sharp angles created artificially by the process of substituting the geometric model by the mesh, reducing artificial peak stresses by improving the representation of the actual geometry. The used mesh density (number of nodes and elements) in each component is given in Table 2 .

**Table 2:** Mesh density

	Model #1: window		Model #2: wrap around	
	Number of nodes	Number of elements	Number of nodes	Number of elements
Cortical bone	63,460	37,503	63,460	37,503
Cancellous bone	113,632	78,929	113,632	78,929
Periodontal ligament	22,960	11,556	22,960	11,556
Tooth structure	164,641	114,943	166,534	116,526
Resin cement	27,941	13,620	43,736	21,494
Veneer layer	27,349	13,383	42,105	20,801

The highest plane of the model was considered fixed in the three directions as a boundary condition. The applied loads were set as 50N, directed with 135° oblique angle from the vertical plane to the following points:

- lingual slope of incisal edge, and
- the junction between incisal and middle thirds.

Total 1212 linear static analyses were performed on a personal computer (Intel Core i7 processor, 2.4 GHz, 6.0 GB RAM), using commercial multipurpose finite element software package (ANSYS version 16.0).

## Results

**Fig. 2:**
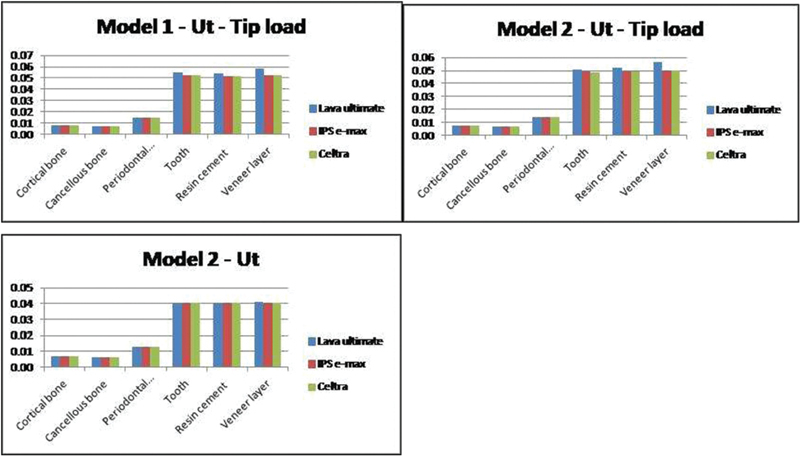
Comparison of total deformation appeared on each model component.

Minor or negligible differences of total deformation were recorded by changing loading position and/or the preparation type on bone and PDL. Total deformation and von Mises stresses on bone (cortical and cancellous) and PDL showed nearly the same values according to the preparation type and applied loading condition (see Fig. 2 ).

The two models' total deformations comparison in Fig. 2 showed a slight increase in tooth structure, cement layer, and veneer layer with Lava Ultimate in comparison to the other two materials. Equivalent values of total deformation were recorded with IPS e-max and Celtra in all cases.

**Fig. 3:**
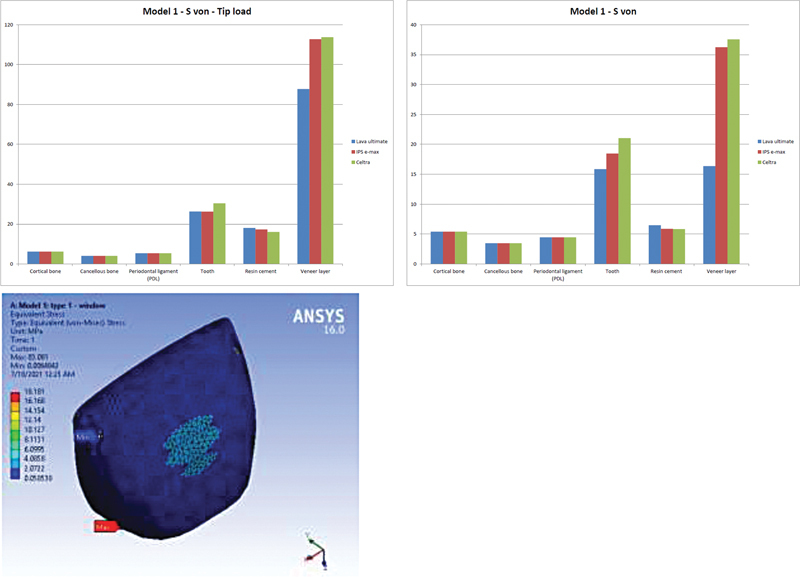
Von Mises stress comparison on Model #1, and example of IPS e-max veneer layer under tip loading.

In model 1 ( Fig. 3 ), finish line resists veneer layer movement under tip loading; thus, it received maximum values of von Mises stress with relatively high values. On the contrary, junction loading creates much lower values of stresses (approximately 30%) in comparison to tip loading.

The general trends of total deformation and von Mises stress in all model components did not change even with changing loading positions. More stiff (or rigid) veneer layer caused and received more stresses on tooth structure, cement, and veneer layers.

**Fig. 4:**
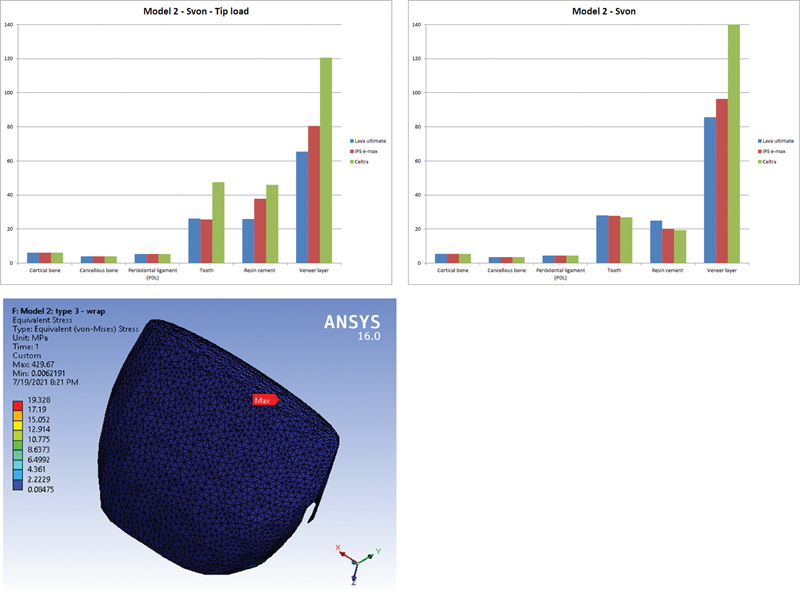
Von Mises stress comparison on Model #2, and sample of cement layer von Mises stress under Celtra veneer layer.

Although the increase of finish line contour in model 2 (wrap around) in comparison to model 1 (window) showed higher stresses, the increase of finish line moved the extreme stress to the new location such that extreme von Mises stress values appeared under the applied loads. The wrap around preparation directly received the applied load at the junction between incisal and middle thirds; thus, it received higher stress in comparison to the window type that did not receive this load ( Fig. 4 ).

Cement layer received higher deformations and stresses under veneer layer prepared as wrap around, transferring the load to the tooth structure, where it received loading via veneer in the two load cases.

## Discussion

Veneers are considered a successful type of restorations as stated by the systematic review by Aljazairy in 2020, showing their high success rate. 16 The perfect way to test a type of dental restoration is in the oral cavity but clinical studies are time-consuming and are not cost-effective. 17 So finite element analysis is used to evaluate the effect of different veneer preparation designs with different materials for fabrication. The preparation of veneers is an important factor in their success as stated by Linhares et al in 2020 especially with premolars. 18 Other recent case reports showed the satisfaction of the patients with tooth preparation of veneers or even without tooth preparation as shown by Sá et al in 2018. 19 There are different preparation designs for laminate veneers, window, feathered edge, palatal chamfer, and butt joint. 20 Increasing the extension of preparation increases the bonded surface area, which is one of the important points in the veneer survival. The decision was made here to compare the cases with incisal coverage and without incisal coverage. A distinct difference between the four types was not always possible. 21 Also names of the preparation designs were not always the same in all studies. Generally, the failures of cases with incisal coverage were less compared to cases without incisal coverage. 2 Other studies as that by Beier et al in 2012 showed that the failures with the overlap design were more compared to no overlap preparation designs. 22

Bone (cortical and cancellous) and PDL were insensitive to veneer material, while minor or negligible changes were noticed by changing the preparation type or loading position. All structures were included in the study to have more realistic results. 23 Our results regarding the bone were in accordance with Tsouknidas et al in 2020 who found that stresses with veneers were the same as with the natural tooth regarding the supporting structures. 14

Tooth structure, cement, and veneer layers showed equivalent values of total deformation under IPS e-max and Celtra in all loading cases that may be referred to close values of elasticity modulus, while the slight increase was also observed in values under Lava Ultimate that may also be referred to the same reason. This was in accordance with Fernandes et al in 2021 who found that the stresses are nearly the same at the tooth structure regardless of the material of fabrication or the preparation design. However, on the contrary, they found higher stresses in the palatal chamfer on the veneer itself. 13

Window preparation type showed a gradual increase in stresses with increasing veneer layer elasticity (correlated to rigidity) that may be caused by the higher material resistance to deflect under load and transfer load to underneath structures at the veneer layer finish line. This was in accordance with Chai et al in 2021 who found in a photoelastic study that stresses were better when covering the incisal edge with the veneer due to distribution over a wider surface area. 15

Although the increase of finish line contour in model 2 (wrap around) in comparison to model 1 (window) showed higher stresses, this may be referred to the loading transfer mechanism, where the veneer layer is floating on (supported by) weaker material (cement layer) that allows veneer layer micro-movement. In addition to having direct contact with loading at junction between incisal and middle thirds, this was in accordance with Fernandes et al in 2021who found that in the case of wrap around the veneers are more susceptible to fracture as stress concentration occurs at the tooth restoration interface at the junction between incisal and middle thirds.

As the cement layer covers, the tooth under veneer works like a cushion to reduce deformation and stresses on the tooth structure. This is why it receives more stresses and deformations under wrap around veneer that it was loaded via veneer layer in both loading cases. This also supported by Li et al in 2014 finite element study who found the same with incisal coverage but under loading with little load angulation 60°. 5

More studies are recommended to evaluate the effect of other materials used for the construction of veneers. In our study, the null hypothesis was proved.

## Conclusions

Bone (cortical and cancellous) and PDL are insensitive to veneer material, while minor or negligible changes may be noticed by changing preparation type or loading position. The veneer layer finish line and its contact with cement layer and tooth structure play a crucial role in the loading transfer mechanism. Thus, the preparation type alters the values of stresses on tooth structure, cement, and veneer layers.

With window preparation type, extreme stresses appear at finish line, while they appeared under the loading site with wrap around preparation. Veneer and cement layer withstand the majority of load energy with wrap around preparation and reduce tooth structure stresses. As deformations and stresses are within physiological limits, the lifetime of veneer and cement layers might be longer with window preparation. This finite element study gives guidelines for operators.
